# Supplementary material for: Structural analysis of 3’UTRs in insect flaviviruses reveals novel determinants of sfRNA biogenesis and provides new insights into flavivirus evolution
Source: Nat Commun. 2022 Mar 11;13:1279. doi: 10.1038/s41467-022-28977-3 (PMC8917146; doi:10.1038/s41467-022-28977-3)
Supplement: Supplementary file 1 — Supplementary Information [file 41467_2022_28977_MOESM1_ESM.pdf]

Supplementary information
Structural analysis of 3'UTRs in insect flaviviruses reveals novel determinants of sfRNA biogenesis and provides new insights into flavivirus evolution

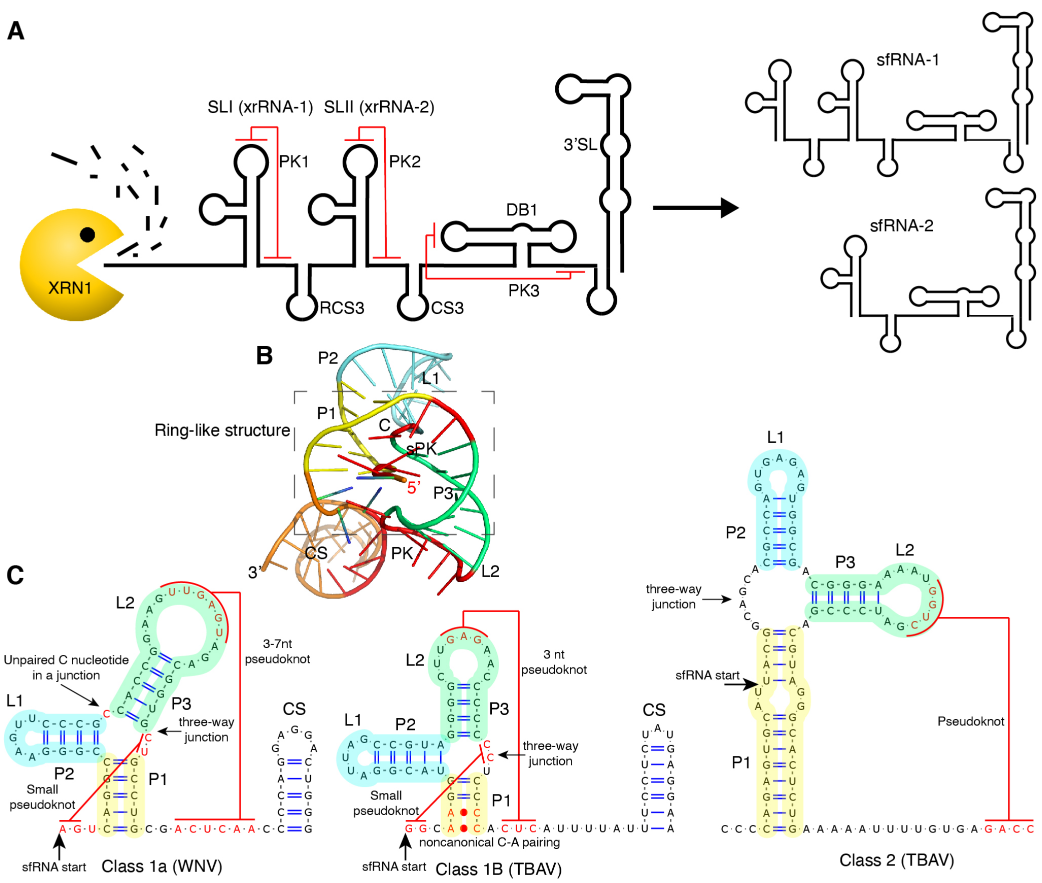

Supplementary figure 1. Known structural determinants of sfRNA biogenesis. (A) Schematic representation of 3'UTR organisation in mosquito-borne flaviviruses (ZIKV) and biogenesis of sfRNAs via XRN1 resistance mechanism. Conserved structural elements of MBF 3'UTRs are labelled. SL – stem-loop, DB – dumbbell, PK – pseudoknot, CS3 and RCS3 – conserved small stem-loops. **(B)** Crystal structure of MVEV xrRNA [PDB ID: 4PQV (https://www.rcsb.org/structure/4pqv)][1] shown as an example of flavivirus xrRNA tertiary structure with a 5'-end of RNA passing through ring-like element. **(C)** Secondary structure of different classes of flavivirus xrRNAs. Secondary structure of WNV xrRNA-1[2], TBAV xrRNA[3] and TBEV xrRNA-1[4] are shown as representative examples of class 1a, class 1b and class 2 xrRNAs, respectively. Functionally important structural elements are indicated and shown in colour. Colours used for highlighting match (B) to show the position of respective RNA helices and pseudoknots. In (B, C) P1-P3 – RNA helices, L1,2 – loops, CS – conserved small stem-loop.

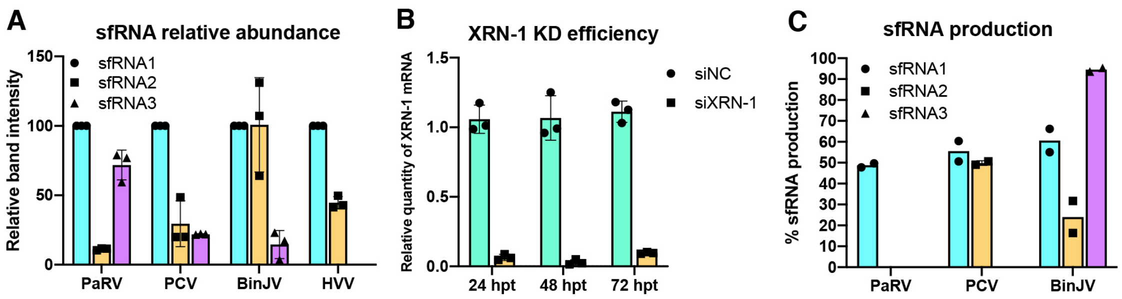

Supplementary figure 2. Quantitative analysis of sfRNA production by ISFs. (A) Relative abundance of the individual sfRNA species produced by ISFs. Abundance was determined as the intensity of the bands (mean gray value) on Northern blots (Fig 1A) and normalised to the intensity of the sfRNA-1 band for each virus, which was considered 100%. **(B)** The XRN1 knock-down efficiency in Aag2 cells transfected with dsRNA for XRN-1 (siXRN-1). Cells were transfected with siXRN-1 or GFP dsRNA (siNC), and RNA was isolated at the indicated time points. Expression of XRN-1 gene was determined by qRT-PCR using ddCT method and presented relative to XRN-1 expression in untransfected cells after normalisation to RPL11 mRNA level. **(C)**

Effect of XRN-1 knock-down on the production of sfRNAs in ISF-infected Aag2 cells. Band intensity on Northern blots (Fig 1B) was determined using digital densitometry, and the percentage of sfRNA production in cells transfected with dsRNA for XRN1 relative to those transfected with GFP dsRNA was calculated. The values are the means of three (A, B) or two (C) independent experiments with error bars indicating for SD **(A, B)** or SEM **(C).**

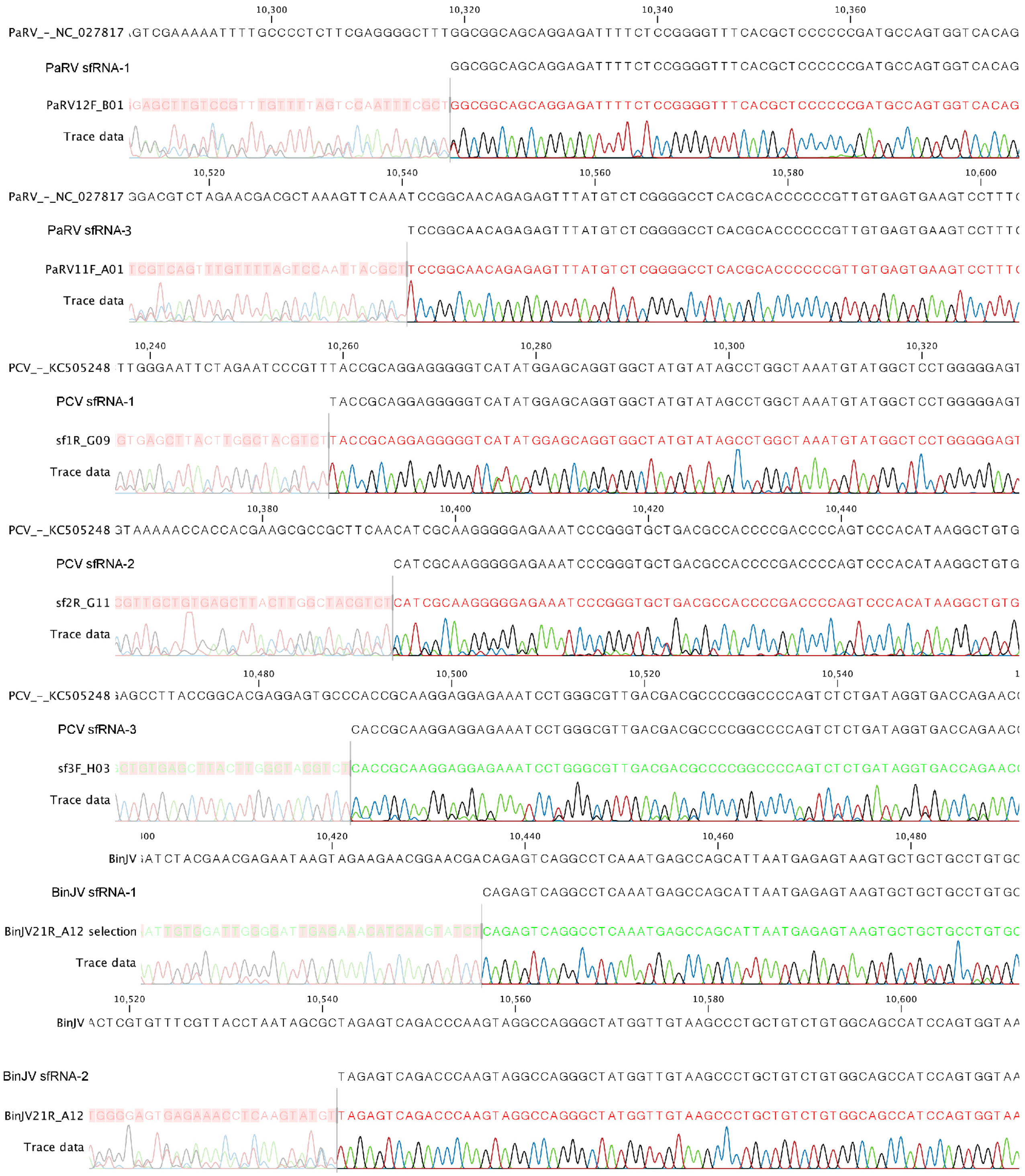

Supplementary figure 3. Sequence alignments showing 5'-ends of ISF sfRNAs. C6/36 cells were infected with PaRV, PCV or BinJV at MOI=1, and total RNA was isolated at 7dpi. RNA was then incubated with RNA-ligase I, which led to the circularisation of 5'-monophosphorylated uncapped RNA (sfRNA) but not capped viral genomic RNA. After ligation reaction, RNA was used for first-strand cDNA synthesis with a reverse primer designed to the 3'-end of viral UTR, followed by PCR amplification with back-to-back primers designed within the last 100nt of 3'UTRs. PCR products were gel-purified, and Sanger sequenced. The resulting sequences were aligned to genomic reference sequences, and 5'-ends of sfRNAs were identified as the first nucleotides downstream of the known 3'-terminal nucleotides in the amplicon's circularised molecules.

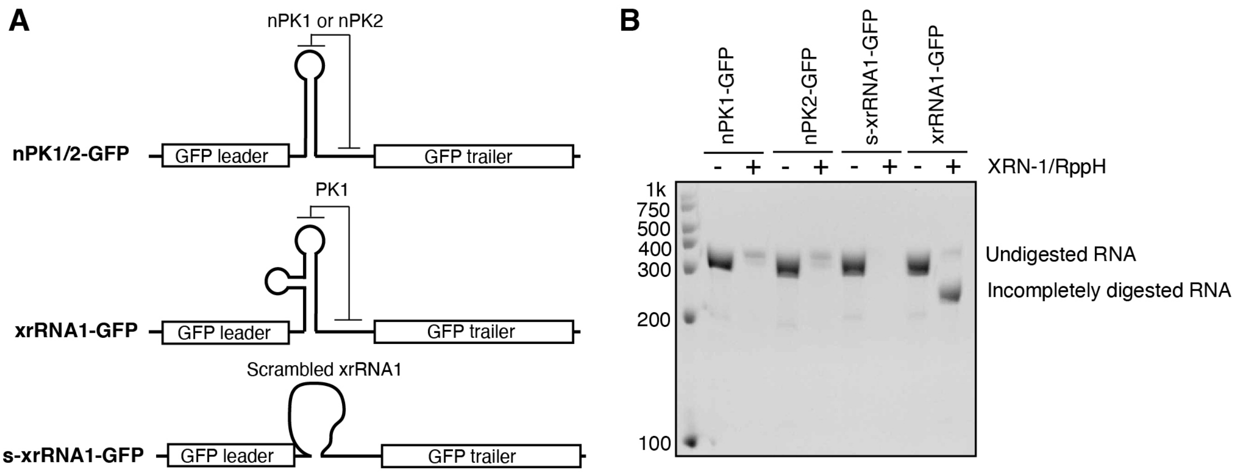

Supplementary figure 4. XRN1 resistance assay of BinJV novel pseudoknots. (A) Schematic representation of the RNA constructs used for assessing the XRN1 resistance of novel pseudoknots (nPKs) of BinJV. **(B)** *In vitro* XRN1 resistance assay with RNA constructs containing novel PKs or conventional xrRNA of BinJV. RNA constructs shown in **(A)** were *in vitro* transcribed and refolded at 28C. Samples were then treated purified XRN1 and RppH and analysed by electrophoresis in denaturing PAAG. Gels were stained with ethidium bromide. The experiment was repeated twice with similar results.

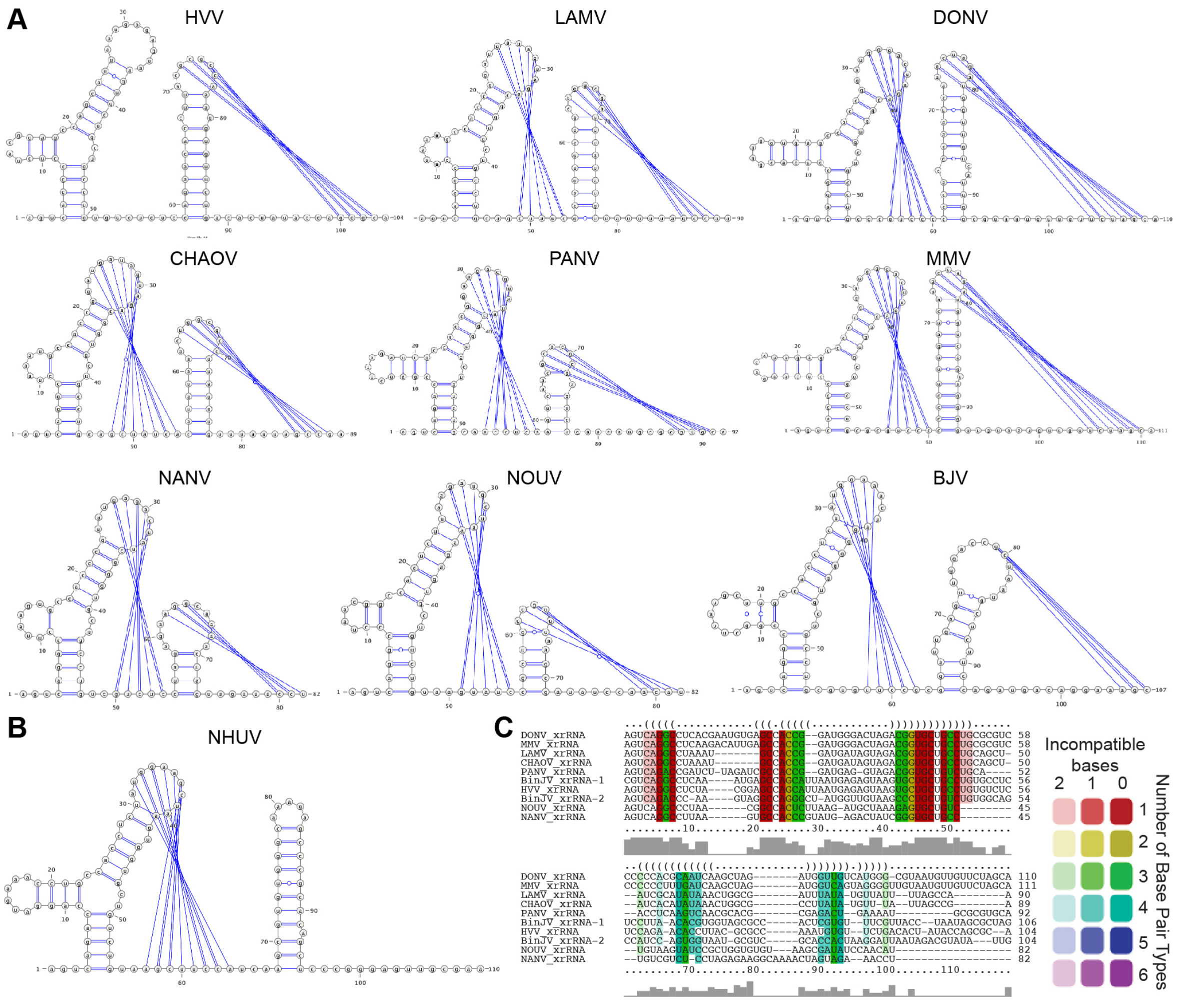

Supplementary figure 5. Conserved secondary structures of dISF xrRNAs. (A) Predicted secondary structures of dISF xrRNAs that form novel PK. **(B)** Predicted secondary structure of Nhumirim virus. In (A, B), putative xrRNAs were located in 3'UTRs of dISFs based on sequence homology with BinJV xrRNAs. Secondary structures and pseudoknots were predicted using the IPknot web server. **(C)** Alignment of sequence and structure of dISF 3'UTR regions containing classical and novel xrRNAs. Structural constraints for alignments were specified based on experimental data (BinJV) or IP-knot prediction (other dISFs).

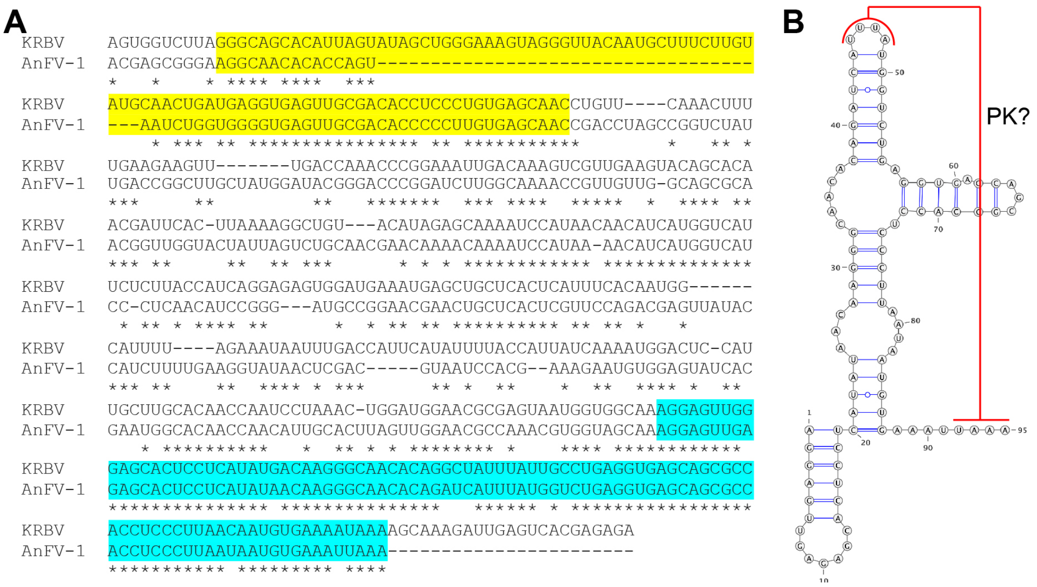

Supplementary figure 6. Regions of homology in 3'UTRs of *Anopheles*-associated ISFs. (A) Sequence alignment of KRBV and AnFV-1 3'UTRs. Regions corresponding to xrRNA are highlighted in yellow, and regions of 3'-terminal SL are shown in cyan. Asterisks indicate identical nucleotides. **(B)** Predicted secondary structure of AnFV-1 3'-terminal stem-loop element.

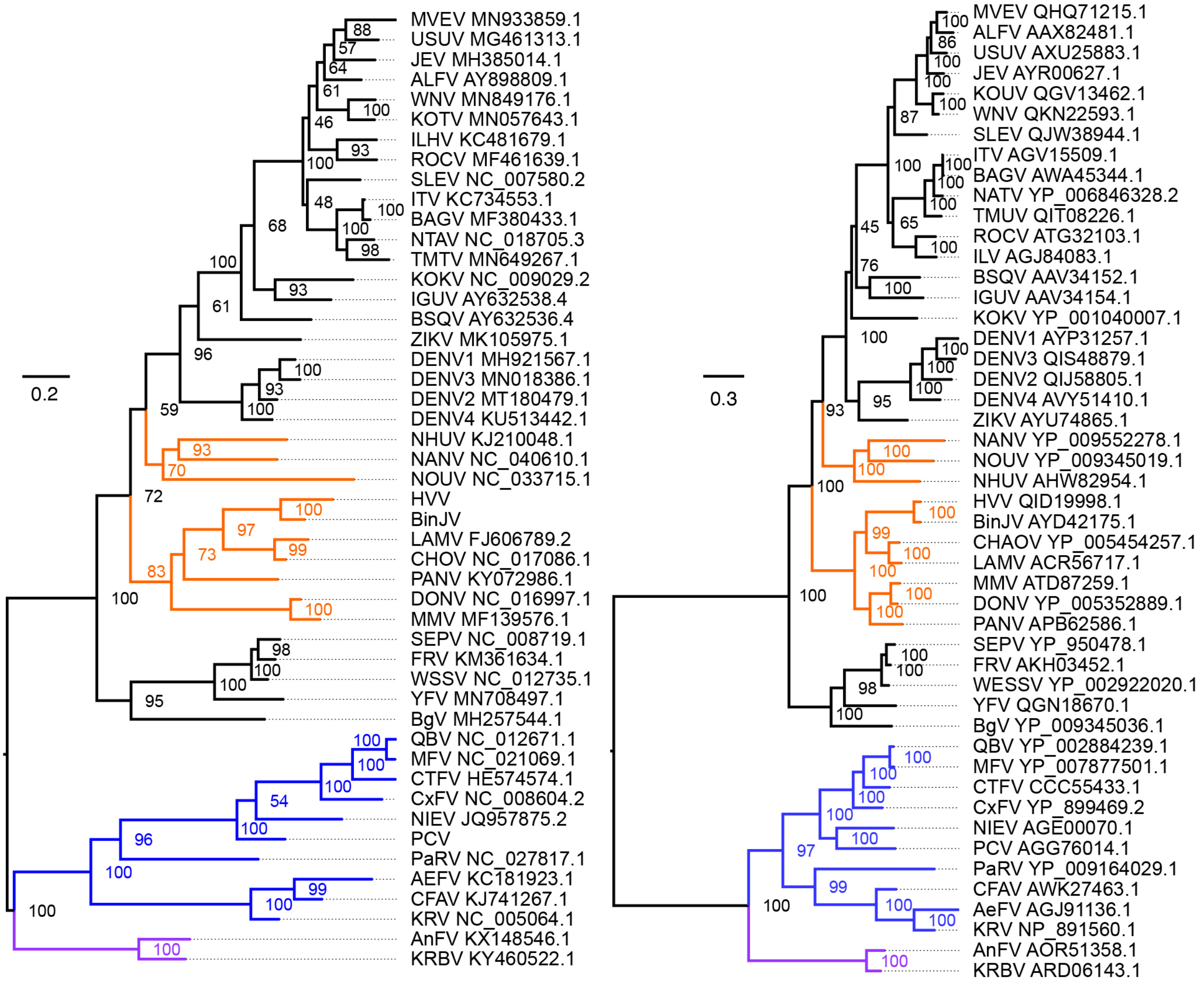

Supplementary figure 7. Trees of phylogenetic relationships between flaviviruses built based on sequence and structure of 3'UTRs (left) and polyprotein sequence (right). MBF clades are shown in black, dISF – in orange, cISF – in blue and *Anopheles*-associated cISFs are shown in purple. Both trees are consensus maximum-likelihood trees constructed using IQ-TREE2 and are midpoint rooted. Numbers at the nodes show bootstrap support.

Supplementary Table 1. Virus acronym, names and Accession IDs for constructing multiple sequence alignment of flavivirus polyprotein and 3'UTRs.

Clade	Virus	Virus name	GenbankID 3'UTR	GenbankID
Anopheles cISF	AnFV	Anopheles flavivirus	KX148546.1	AOR51358.1
	KRBV	Karumba virus	KY460522.1	ARD06143.1
cISF	AeFV	Aedes flavivirus	KC181923.1	AGJ91136.1
	CFAV	Cell fusing agent virus	MH237596.1	AWK27463.1
	CTFV	Culex theileri flavivirus	HE574574.1	CCC55433.1
	CxFV	Culex flavivirus	NC_008604.2	YP_899469.2
	KRV	Kamiti River virus	NC_005064.1	NP_891560.1
	MFV	Mosquito flavivirus	NC_021069.1	YP_007877501.1
	NIEV	Nienokoue virus	JQ957875.2	AGE00070.1
	PaRV	Parramatta River virus	NC_027817.1	YP_009164029.1
	PCV	Palm Creek virus	This study	AGG76014.1
	QBV	Quang Binh virus	NC_012671.1	YP_002884239.1
dISF	BinJV	Binjari virus	This study	AYD42175.1
	CHAOV	Chaoyang virus	NC_017086.1	YP_005454257.1
	DONV	Donggang virus	NC_016997.1	YP_005352889.1
	HVV	Hidden valley virus	This study	QID19998.1
	LAMV	Lammi virus	FJ606789.2	ACR56717.1
	MMV	Marisma mosquito virus	MF139576.1	ATD87259.1
	NANV	Nanay virus	NC_040610.1	YP_009552278.1
	NHUV	Nhumirim virus	KJ210048.1	AHW82954.1
	NOUV	Nounane virus	NC_033715.1	YP_009345019.1
	PANV	Panmunjeom flavivirus	KY072986.1	APB62586.1
MBF	ALFV	Alfuy virus	AY898809.1	AAX82481.1
	BAGV	Bagaza virus	MF380433.1	AWA45344.1
	BgV	Bamaga virus	MH257544.1	YP_009345036.1
	BSQV	Bussuquara virus	AY632536.4	AAV34152.1
	DENV1	Dengue fever virus 1	MH921567.1	AYP31257.1
	DENV2	Dengue fever virus 2	MT180479.1	QIJ58805.1
	DENV3	Dengue fever virus 3	MT261978.1	QIS48879.1
	DENV4	Dengue fever virus 4	MF004387.1	AVY51410.1
	FRV	Fitzroy River Virus	KM361634.1	AKH03452.1
	IGUV	Iguape virus	AY632538.4	AAV34154.1
	ILHV	Ilheus virus	KC481679.1	AGJ84083.1
	ITV	Israel turkey meningoencephalomyelitis virus	KC734553.1	AGV15509.1
	JEV	Japanese encephalitis virus	MH385014.1	AYR00627.1
	KOKV	Kokobera virus	NC_009029.2	YP_001040007.1
	KOUV	Koutango virus	MN057643.1	QGV13462.1
	MVEV	Murray Valley encephalitis virus	MN933859.1	QHQ71215.1
	NTAV	Ntaya virus	NC_018705.3	YP_006846328.2
	ROCV	Rocio virus	MF461639.1	ATG32103.1
	SEPV	Sepik virus	NC_008719.1	YP_950478.1
	SLEV	Saint Louis encephalitis virus	MN233334.1	QJW38944.1
	TMUV	Tembusu virus	MN649267.1	QIT08226.1
	USUV	Usutu virus	MG461313.1	AXU25883.1
	WESSV	Wesselsbron virus	NC_012735.1	YP_002922020.1
	WNV	West Nile virus	MN849176.1	QKN22593.1
	YFV	Yellow fever virus	MN708497.1	QGN18670.1
	ZIKV	Zika virus	MK105975.1	AYU74865.1

Supplementary Table 2. Oligonucleotides used in the study.

**Northern Blotting probes**	
PaRV NB Probe	AGCGTAATTGGACTAAAACAAACTG
PCV NB probe	AGACGTAGCCAAGTAAGCTCACAG
KRBV NB Probe	GTTAAGGGAGGTGGCGCTGCTCACC
BinJV NB Probe	AGATACTTGATGTTTCTCAATC
HVV NB Probe	ACTTGATGTTTCTCAATTCCCAATCCAC
**sfRNA Sequencing Primers**	
KRBV_Lig_Seq_F	GCGAGTAATGGTGGCAAAGGAGTTGGG
KRBV_Lig_seq_F	GTTCCATCCAGTTTAGGATTGGTTGTGCAAGC
KRBV_Lig_Seq_RT	TCTCTCGTGACTCAATCTTTGCTTTTATTTTCACATTGTTAAGGG
PCV_Lig_Seq_F	GGGCTTAGCCCAAGGTGAGTGACGA
PCV_Lig_Seq_R	TGTGTGCCCTCACCATTTCTGGTGATC
PCV_Lig_Seq_RT	AGACGTAGCCAAGTAAGCTCACAGCAACG
PaRV_Lig_Seq_F	CCCGGTTGTGAAAACGATTGCGACTAGAA
PaRV_Lig_Seq_R	GGGTGACGCTACTCACCCTAGTTCGTT
PaRV_Lig_Seq_RT	AGCGTAATTGGACTAAAACAAACTGACGAGCTCC
BinJV-Lig_Seq_RT	AGATACTTGATGTTTCTCAATCCCCAATCCACAATTATTCGG
BinJV_Lig_Seq_F	CGACACCTGGGAAAGACCGGAGATACC
BinJV_Lig_Seq_R	ATATGATGCTCTACATTTTGGAGGGGGTTTCCTCT
**Primers for generation of IVT template for XRN-1 siRNA**	
T7-Aedes_XRN1-1s-F	TCGCAGtaatacgactcactatagggCGAGTTGCCGAAGGAGCCGT
T7-Aedes_XRN1-1s-R	GCGGAACATTGGGCGGATGC
T7-Aedes_XRN1-1a-F	CGAGTTGCCGAAGGAGCCGT
T7-Aedes_XRN1-1a-R	TCGCAGtaatacgactcactatagggGCGGAACATTGGGCGGATGC
T7-Aedes_XRN1-2s-F	TCGCAGtaatacgactcactatagggGCCCCATCTAACCGAGGCCA
T7-Aedes_XRN1-2s-R	CCGATCGTCTCGCTCCTCGC
T7-Aedes_XRN1-2a-F	GCCCCATCTAACCGAGGCCA
T7-Aedes_XRN1-2a-R	TCGCAGtaatacgactcactatagggCGAGTTGCCGAAGGAGCCGT
**3'UTR cloning primers**	
PaRV-3UTR T7 F	TAATACGACTCACTATAGGGgaaaccatcttttccaaattag
PaRV-3UTR T7 R	AGCGTAATTGGACTAAAACAAACTGACGAGC
PCV-3UTR T7 F	TAATACGACTCACTATAGGGgaaaaatccttgagcaggag
PCV-3UTR T7 R	AGACGTAGCCAAGTAAGCTCACAGCAACGC
BinjV-3UTR T7 F	TAATACGACTCACTATAGGGggatctacgaacgagaataag
BinjV-3UTR T7 R	AGATACTTGATGTTTCTCAATCCCCAATCCACAATT
KRBV-3UTR T7 F	TAATACGACTCACTATAGGGccaaattacgacgtgtcttgg
KRBV-3UTR T7 R	TCTCTCGTGACTCAATCTTTGCTTTTATTTTCACATTGTTAAGG
**SHAPE Primers**	
PaRV_233-FAM	/56-FAM/CTAGACGTCCTTCGAAACCAGTTCG
PCV_243-FAM	/56-FAM/CGTCACAGCCTTATGTGGGACTGGG
BinJV_379-FAM	/56-FAM/TGTGCCTTGTGGATTGAGTGCTGTG
BinJV_200-FAM	/56-FAM/CGACGCATTACCACTGGATGGCUG
KRBV_379-FAM	/56-FAM/AGTGCTCCCAACTCCTTTGCCACC
KRBV_447-FAM	/56-FAM/CATTGTTAAGGGAGGTGGCGCTG
HVV_187-FAM	/56-FAM/CGGGCGCCATTCCACTATGAGGTTG
PaRV_233-HEX	/5HEX/CTAGACGTCCTTCGAAACCAGTTCG
PCV_243-HEX	/5HEX/CGTCACAGCCTTATGTGGGACTGGG
BinJV_379-HEX	/5HEX/TGTGCCTTGTGGATTGAGTGCTGTG
BinJV_200-HEX	/5HEX/CGACGCATTACCACTGGATGGCUG
KRBV_379-HEX	/5HEX/AGTGCTCCCAACTCCTTTGCCACC
KRBV_447-HEX	/5HEX/CATTGTTAAGGGAGGTGGCGCTG
HVV_187-HEX	/5HEX/CGGGCGCCATTCCACTATGAGGTTG
**3'UTR sequencing Primers**	
PaRV_3UTR_Seq F	AGAGACTTGGAGTTGTGGCG
PaRV_3UTR_Seq R	AGCGTAATTGGACTAAAACAAACTG
PCV_3UTR_Seq_F	AGTGTGACGATGTAGGCCCG
PCV_3UTR_Seq_R	AGACGTAGCCAAGTAAGCTCACAGC
**Mutagenesis Primers**	
PCV_PK1'F	CTGGGGGAGTctgGCCCCTCCGG
PCV_PK1'R	GAGCCATACATTTAGCCAGG
PCV_PK2'_F	CCCGGGTGCTctgGCCACCCCGA
PCV_PK2'_R	ATTTCTCCCCCTTGCGATGTTG
PCV_PK3'_F	CCTGGGCGTTctgGACGCCCCGG
PCV_PK3'_R	ATTTCTCCTCCTTGCGGTGG
PaRV_PK1'_F	TCCGGGGTTTgtgGCTCCCCCCG
PaRV_PK1'_R	GAAAATCTCCTGCTGCCG
PaRV_PK2'_F	CTCGGGGATTgtgGCTCCCCCCAT
PaRV_PK2'_R	ACAAGCTCTGCTGCCATA
PaRV_PK3'_F	CTCGGGGCCTgtgGCACCCCCCG
PaRV_PK3'_R	ACATAAACTCTCTGTTGCCGGATTTG
KRBV_PK1'_F	GATGAGGTGAcaacgGACACCTCCCTGTGAGC
KRBV_PK1'_R	AGTTGCATACAAGAAAGC
BinJV PK1' F	CAGCATTAATCTCTCTAAGTGCTGCTGCCTGTG

BinjJV PK1' R	GCTCATTTGAGGCCTGAC
BinJV PK2' F	CCAGGGCTATCCAACTAAGCCCTGCTG
BinJV_PK3' F	GGAGCAGCAAcgagGAGCTGCATCACCCAC
BinJV_PK3' R	TCACCAGTCCCTCCCAAT
BinJV PK3' R	CCTCACCAGTCCCTCCCA
BinJV nPK1' F	AACACGTGGTTCGCCCACTCGTGTTTCG
BinJV nPK1' R	AAGGAGAGGCACAGGCAG
BinJV_nPK2'F	CAGTGGTAATCGCAGGCACCACTAAGGATTAATAGACG
BinJV_nPK2'R	GATGGCTGCCACAGACAG
**CPER primers**	
ParV_linkerF	CAGTTTGTTTTAGTCCAATTACGCTGGGTCGGCATGGCATCTCCAC
ParV_linkerR	AACCACGGGTTAACTTTTAAAAACTGTTTACCAGATCGTTGCGGGC
ParV_frag1F	AGTTTTTAAAAGTTAACCCGTGGTTTTACC
ParV_frag1R	AACCGGTTTGTTCTCCCTAGTC
ParV_frag2F	GACTAGGGAGAACAAACCGGTT
ParV_frag2R	GTATGCCCTCATTACTAGCCCC
ParV_frag3F	GGGGCTAGTAATGAGGGCATAC
ParV_frag3R	CCTTCTGGCTTCCAACCATACT
ParV_frag4F	AGTATGGTTGGAAGCCAGAAGG
ParV_frg4R	AGCGTAATTGGACTAAAACAAACTGA
PCV_linker_F	GTGAGCTTACTTGGCTACGTCTGGGTCGGCATGGCATCTCCAC
PCV_linker_R	ACTAACGCAAAAGTTTTTTAAAACTGTTTACCAGATCGTTGCGGGC
PCV_frag1_F	AGTTTTAAAAAACTTTTGCGTTAGT
PCV_frag1_R	ACTCCTCTCGAACTCTCCATCA
PCV_frag2_F	TGATGGAGAGTTCGAGAGGAGT
PCV_frag2_R	CTCCCAAAGTCACCCCGATAAA
PCV_frag3_F	TTTATCGGGGTGACTTTGGGAG
PCV_frag3_R	TAACGTCAGTGGATGGAAGTGG
PCV_frag4_F	CCACTTCCATCCACTGACGTTA
PCV_frag4_R	AGACGTAGCCAAGTAAGCTCACAG
BinJV_5'-E_F	AACGATCTGGTAAACAGTATATTTTGCGTG
BinJV_5'-E_R	TCCTATTTCCGATAGGGCACCCACGGTCAC
BinJV_1-2B_F	GTGACCGTGGGTGCCCTATCGGAAATAGGA
BinJV_1-2B_R	CCACAACACAGTCCCCCGCTTGTTTGATTT
BinJV_3-4B_F	AAATCAAACAAGCGGGGGACTGTGTTGTGG
BinJV_3-4B_R	GGTGGCCTGTAATCCCCTCCTAGGAACTCC
BinJV_NS5-3'_F	GGAGTTCCTAGGAGGGGATTACAGGCCACC
BinJV_NS5-3'Junc_R	CTCGTTCGTAGATCCTTAGATCACATTGCC
BinJV_NS5-3'Junc_F	GGCAATGTGATCTAAGGATCTACGAACGAG
BinJV_NS5-3'_R	TGCCATGCCGACCCAGATACTTGATGTTTC
BinJV3'UTR-linker	TGGATTGGGGATTGAGAAACATCAAGTATCTGGGTCGGCATGGCATCTCCACCTCCTCGC
Linker-BinJV5'UTR	GTGTTTTGAAACGCACACGCAAAATATACTGTTTACCAGATCGTTGCGGGCTGTATTTATAGGC

References

1. Chapman, E. G. *et al.* The structural basis of pathogenic subgenomic flavivirus RNA (sfRNA) production. *Science.* **344**, 307–310 (2014).
2. Funk, A. *et al.* RNA structures required for production of subgenomic flavivirus RNA. *J. Virol.* **84**, 11407–17 (2010).
3. Jones, R. A. *et al.* Different tertiary interactions create the same important 3D features in a distinct flavivirus xrRNA. *RNA.* **27**, 54–65 (2021).
4. MacFadden, A. *et al.* Mechanism and structural diversity of exoribonuclease-resistant RNA structures in flaviviral RNAs. *Nat. Commun.* **9**, 119 (2018).
