# Supplementary material for: Structural analysis of 3’UTRs in insect flaviviruses reveals novel determinants of sfRNA biogenesis and provides new insights into flavivirus evolution
Source: Nat Commun. 2022 Mar 11;13:1279. doi: 10.1038/s41467-022-28977-3 (PMC8917146; doi:10.1038/s41467-022-28977-3)
Supplement: Supplementary file 3 — Reporting Summary [file 41467_2022_28977_MOESM3_ESM.pdf]

nature portfolio

Corresponding author(s): Alexander A Khromykh, Andrii Slonchak
Last updated by author(s): Feb 1, 2022

Reporting Summary

Nature Portfolio wishes to improve the reproducibility of the work that we publish. This form provides structure for consistency and transparency in reporting. For further information on Nature Portfolio policies, see our Editorial Policies and the Editorial Policy Checklist.

Statistics

For all statistical analyses, confirm that the following items are present in the figure legend, table legend, main text, or Methods section.

n/a	Confirmed	
☐	☒	The exact sample size (n) for each experimental group/condition, given as a discrete number and unit of measurement
☒	☐	A statement on whether measurements were taken from distinct samples or whether the same sample was measured repeatedly
☐	☒	The statistical test(s) used AND whether they are one- or two-sided<br>*Only common tests should be described solely by name; describe more complex techniques in the Methods section.*
☒	☐	A description of all covariates tested
☒	☐	A description of any assumptions or corrections, such as tests of normality and adjustment for multiple comparisons
☐	☒	A full description of the statistical parameters including central tendency (e.g. means) or other basic estimates (e.g. regression coefficient) AND variation (e.g. standard deviation) or associated estimates of uncertainty (e.g. confidence intervals)
☐	☒	For null hypothesis testing, the test statistic (e.g. F, t, r) with confidence intervals, effect sizes, degrees of freedom and P value noted<br>*Give P values as exact values whenever suitable.*
☒	☐	For Bayesian analysis, information on the choice of priors and Markov chain Monte Carlo settings
☒	☐	For hierarchical and complex designs, identification of the appropriate level for tests and full reporting of outcomes
☒	☐	Estimates of effect sizes (e.g. Cohen's d, Pearson's r), indicating how they were calculated

Our web collection on statistics for biologists contains articles on many of the points above.

Software and code

Policy information about availability of computer code

Data collection	Image Studio Lite software v5.2.5 (LI-COR, USA)
Data analysis	ImageJ2 Fiji v.2019.10.27 (https://fiji.sc)<br>IPknot v1.4.1. (http://rtips.dna.bio.keio.ac.jp/ipknot/)<br>mfold v2.3 (http://www.unafold.org/mfold/applications/rna-folding-form-v2.php)<br>VARNA v3.93 (http://varna.lri.fr)<br>LocARNA v1.9.1 (http://rna.informatik.uni-freiburg.de/LocARNA/)<br>R-scape v1.4.0 (Eddy Lab, http://eddylab.org/R-scape/)<br>MAFFT v7.475 (https://mafft.cbrc.jp/alignment/software/)<br>IQ-TREE2 v2.1.2 (http://www.iqtree.org)<br>FigTree v1.4.4 (https://github.com/rambaut/figtree/)<br>QuShape v1.0 (https://weekslab.com/software/qushape/)<br>RNAStructure v6.4 (Mathews lab, https://rna.urmc.rochester.edu/RNAstructure.html)<br>Prism v9.0 (Graph Pad Inc)

For manuscripts utilizing custom algorithms or software that are central to the research but not yet described in published literature, software must be made available to editors and reviewers. We strongly encourage code deposition in a community repository (e.g. GitHub). See the Nature Portfolio guidelines for submitting code & software for further information.

Data

Policy information about availability of data

All manuscripts must include a data availability statement. This statement should provide the following information, where applicable:

- Accession codes, unique identifiers, or web links for publicly available datasets
- A description of any restrictions on data availability
- For clinical datasets or third party data, please ensure that the statement adheres to our policy

The SHAPE data generated in this study have been deposited in the Figshare database under accession code 19100390 [https://doi.org/10.6084/m9.figshare.19100390]. The raw gel quantification and virus titration data generated in this study are provided in the Source Data file. All other data are available within the paper and supplementary materials.

Field-specific reporting

Please select the one below that is the best fit for your research. If you are not sure, read the appropriate sections before making your selection.

☒ Life sciences ☐ Behavioural & social sciences ☐ Ecological, evolutionary & environmental sciences

For a reference copy of the document with all sections, see nature.com/documents/nr-reporting-summary-flat.pdf

Life sciences study design

All studies must disclose on these points even when the disclosure is negative.

Sample size	No sample size calculations were performed. All experiments involving statistical analysis were performed at least three times which is the number of replicates that was commonly used in similar studies before.
Data exclusions	No data was excluded
Replication	All experiments were repeated at least two times and produced similar results
Randomization	Randomization was not required as in in vitro experiments cells with different treatments cannot be randomized
Blinding	Blinding was not possible for the experiments with cell lines performed by the same researcher at all stages. All experiments were designed with the appropriate controls, and samples for comparison were collected and analyzed under the same conditions.

Reporting for specific materials, systems and methods

We require information from authors about some types of materials, experimental systems and methods used in many studies. Here, indicate whether each material, system or method listed is relevant to your study. If you are not sure if a list item applies to your research, read the appropriate section before selecting a response.

Materials & experimental systems

n/a	Involved in the study	
☐	☒	Antibodies
☐	☒	Eukaryotic cell lines
☒	☐	Palaeontology and archaeology
☐	☒	Animals and other organisms
☒	☐	Human research participants
☒	☐	Clinical data
☒	☐	Dual use research of concern

Methods

n/a	Involved in the study	
☒	☐	ChIP-seq
☒	☐	Flow cytometry
☒	☐	MRI-based neuroimaging

Antibodies

Antibodies used	Flavivirus E-protein (4G2) Mouse mAb was generated previously (Gentry et al., 1982) and was obtained from Prof R.A. Hall (University of Queensland).<br>PaRV E-protein (7D11) Mouse mAb was generated previously (Piyasena et al., 2017) and was obtained from Prof R.A. Hall (University of Queensland).<br>PCV E-protein (5G12) Mouse mAb was generated previously (Piyasena et al., 2017) and was obtained from Prof R.A. Hall (University of Queensland).<br>IRDye® 800CW Goat anti-Mouse IgG Secondary Antibody (LI-COR, Cat # P/N: 925-32210)
Validation	Flavivirus E-protein (4G2) Mouse mAb was validated previously to specifically recognize E-proteins of BinJV and HVV:

Hobson-Peters, J. et al. A recombinant platform for flavivirus vaccines and diagnostics using chimeras of a new insect-specific virus. Sci. Transl. Med. 11, 1–16 (2019).
Harrison, J.J. et al. Antigenic Characterization of new lineage II insect-specific flaviviruses in Australian mosquitoes and identification of host restriction factors. mSphere 5, e00095-20 (2020).
7D11 and 5G12 antibodies were validated previously to specifically recognize E-proteins of PaRV and PCV, respectively:
Piyasena, T. B. H. et al. Infectious DNAs derived from insect-specific flavivirus genomes enable identification of pre- and post-entry host restrictions in vertebrate cells. Sci. Rep. 7, 2940 (2017).
IRDye® 800CW Goat anti-Mouse IgG Secondary Antibody was validated by manufacturer. Based on ELISA and flow cytometry, this antibody reacts with the heavy and light chains of mouse IgG1, IgG2a, IgG2b, and IgG3, and with the light chains of mouse IgM and IgA. This antibody was tested by dot blot and and/or solid-phase adsorbed for minimal cross-reactivity with human, rabbit, goat, rat, and horse serum proteins, but may cross-react with immunoglobulins from other species.

Eukaryotic cell lines

Policy information about cell lines

Cell line source(s)	Aedes albopictus larvae cells C6/36 (ATCC – CRL-1660) and Aedes aegypti larvae cells Aag2 (ATCC – CCL-125) were obtained from the ATCC. Culture of Aedes albopictus larvae cells RML-12 (Rocky Mountains Laboratory 12) was established by G Kuno (PMID: 6137452) and cell line was obtained from Prof. Robert Tesh (UTMB, USA).
Authentication	Cell lines were not genetically validated, but their morphologies were visually confirmed.
Mycoplasma contamination	All cells were tested and confirmed mycoplasma-negative
Commonly misidentified lines (See ICLAC register)	No commonly misidentified cell lines were used in the study

Animals and other organisms

Policy information about studies involving animals; ARRIVE guidelines recommended for reporting animal research

Laboratory animals	No laboratory animals were used in the study
Wild animals	No wild animals were used in the study
Field-collected samples	Anopheles sp. mosquitoes were collected collected from the Bradshaw Field Training Area in the Karumba region of Northern Queensland, Australia. Adult mosquitoes were collected using Centers for Disease Control (CDC) light traps (John W. Hock Company, Gainesville, FL) baited with CO_2 (1 kg dry ice) and 1-octen-3-ol (release rate: 4.5 mg/h) operated overnight for 12 h (1800–0600 hours). Mosquitoes were killed in field by placing into liquid nitrogen in which they were kept for transport to the laboratory and further storage at -80°C. Mosquitoes were identified morphologically to species on a refrigerated cold table and sorted into pools of ≤20. Field collected mosquitoes were not housed or maintained alive in any way.
Ethics oversight	The study did not require the ethical approval

Note that full information on the approval of the study protocol must also be provided in the manuscript.
